# Synthesis of N‐Acetyl‐D‐ and ‐L‐Leucine‐^13^C_6_ Tool Compounds in Neurodegenerative Disease

**DOI:** 10.1002/cmdc.70295

**Published:** 2026-05-15

**Authors:** Damien Crepin, Andrew McGown, Dawn Shepherd, Rebecca Braine, Manvendra Sharma, Jordan Nafie, João Gabriel Ribeiro, G. Dan Pantoş, Grant Churchill, Frances M. Platt, John Spencer

**Affiliations:** ^1^ Sussex Drug Discovery Centre School of Life Sciences University of Sussex Falmer UK; ^2^ Department of Chemistry School of Life Sciences University of Sussex Falmer UK; ^3^ Department of Pharmacology University of Oxford Oxford UK; ^4^ BioTools Inc. West Palm Beach FL USA; ^5^ Department of Chemistry University of Bath Bath UK

**Keywords:** amino acid, labelled compound, NPC, tool compound

## Abstract

N‐acetyl‐L‐leucine (levacetylleucine, ALL) is the neuroprotective enantiomer of the racemic antivertigo drug, Tanganil (acetyl‐DL‐leucine, ADLL). ALL has recently been clinically repurposed for the treatment of Niemann Pick disease type C1, a disorder characterised by lysosomal accumulation of glycolipids and cholesterol. Isotopically labelled ALL was required, as well as its negative control, D‐enantiomer (ADL), to monitor the in vivo metabolic fate of the drug and to distinguish it from endogenous leucine and associated metabolites. Here, we describe the synthesis of N‐acetyl‐D‐ and ‐L‐leucine‐^13^C_6_, as well as N‐acetyl‐L‐leucine‐^13^C_1_, by N‐acetylation of their amino acid precursors, their spectroscopic characterisation and stereochemical and stability studies. These investigations found that formation of the products, as well as their water‐soluble sodium salt formulation, occurs without compromising stereochemical integrity.

## Graphical abstract

Isotopically labelled N‐acetyl‐leucine analogues have been made and fully characterised as chemical tools for studying neurodegenerative diseases.

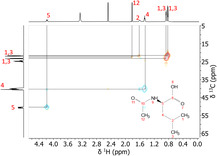

## 1 Introduction

N‐Acetyl‐DL‐leucine (ADLL) has been prescribed for many decades in France for the treatment of ataxia and vertigo, under the brand name Tanganil [1], and has recently been investigated for the treatment of neurological disorders including ataxia, atrophy and REM (Rapid eye movement) sleep behaviour disorder [2, 3, 4, 5, 6]. The individual enantiomers of this derivatised amino acid have distinct roles in disease, with the recently clinically approved ALL (N‐acetyl‐L‐leucine, Aqneursa) [7] offering neuroprotection in the treatment of the rare lysosomal disease, Niemann‐Pick disease type C1 (NPC) [8, 9, 10].

ALL is a prodrug of L‐leucine and is taken up in cells mainly by organic anionic transporters (OAT1 and 3) and monocarboxylate transporter type 1 (MCT1), thereby avoiding the L‐type amino acid transporter used by the free amino acid [11]. L‐leucine plays a vital role in brain energy metabolism. It acts as a source of fuel molecules, including alpha‐ketoisocaproate, maintaining nitrogen balance in the glutamate/glutamine cycle and is key to mTOR (mammalian target of rapamycin) signalling [12]. In vivo metabolism studies of either enantiomer of ADLL would be challenging, as they would be confounded by the presence of indistinguishable endogenous L‐leucine and its metabolites. Therefore, we opted to synthesise N‐acetyl‐L‐leucine‐^13^C_6_ as a labelled form of ALL (with an unlabelled acetamide group), carrying *a* + 6 mass spectrometry ‘tag’ to distinguish it from endogenous amino acid [13, 14, 15, 16, 17, 18, 19, 20, 21, 22]. For comparative studies, a negative control was required, and we synthesised and characterised its ^13^C‐labelled inactive, yet metabolically more stable, ADL enantiomer [23].

## 2 Results and Discussion

**SCHEME 1:**
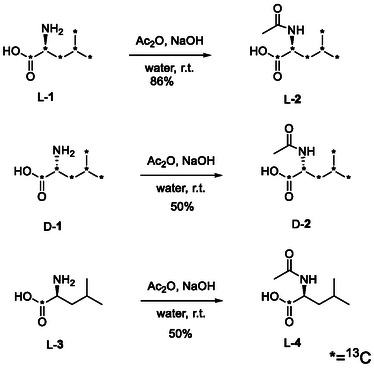
Synthesis of ^13^C labelled ALL and ADL.

N‐acetylation of L‐leucine‐^13^C_6_ L‐**1** was carried out using acetic anhydride in water and the expected N‐acetylated product L‐**2** (ALL‐^13^C_6_) was obtained in 86% yield, on a *ca*. 6 mmol scale. This was repeated on its enantiomer, affording D‐**2** (ADL‐^13^C_6_) in 50% yield, on approximately 1/10^th^ scale (0.69 mmol), both procedures unoptimised due to the prohibitive cost of their precursors. A more economically viable precursor would appear to be the singularly labelled L‐leucine‐^13^C_1_ precursor, **3**, which was similarly converted into its acetylated form L‐**4** in 50% yield (Scheme 1).

**FIGURE 1:**
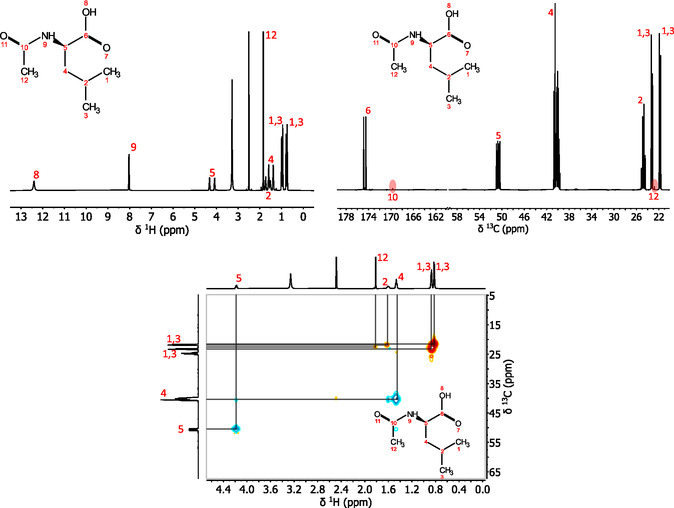
NMR spectra for L‐**2** and peak assignment according to the numbering scheme on the chemical structure. Top trace: ^1^H, ^13^C‐decoupled spectrum (1024 scans). Middle trace: ^13^C{^1^H} spectrum (2048 scans). Bottom: HSQC spectrum (using the hsqcedetgpsisp2.2 pulse programme with 64 scans per increment and 256 increments in the ^13^C dimension).

Mass spectrometry showed the expected isotopic incorporation for L‐ and D‐**2** and for L‐**4** (Experimental Section). The structure of L‐**2** was further confirmed by ^1^H and ^13^C NMR (nuclear magnetic resonance) spectroscopy, and peaks were assigned by the acquisition of ^1^H ^13^C‐decoupled, AMA (automatic multiplet assignment) and an HSQC (heteronuclear single quantum correlation) experiment as well as by comparison to literature NMR data on L‐leucine‐^13^C_6_ (Figure 1) [24].

**FIGURE 2:**
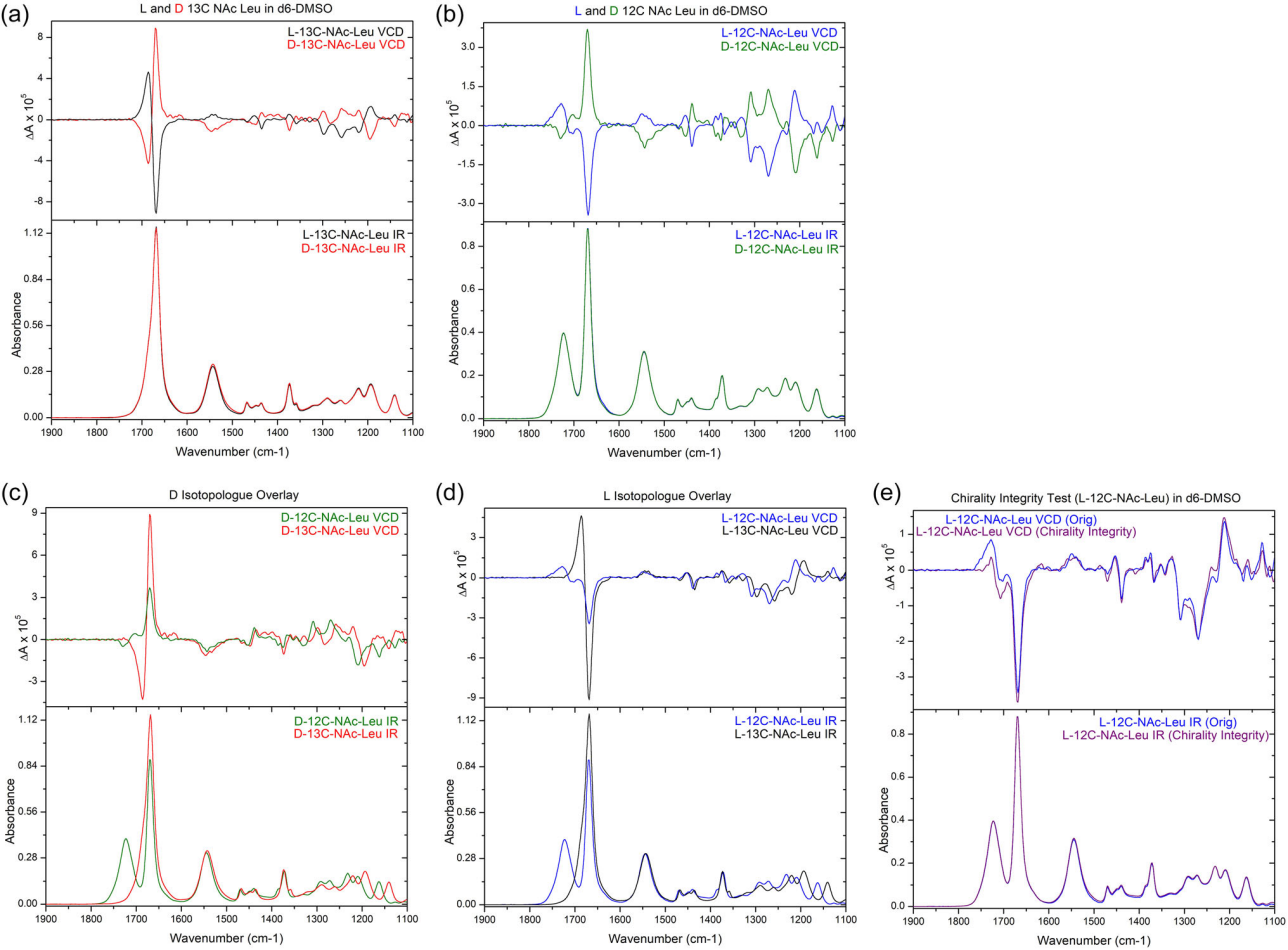
(a) VCD for L‐**2** and D‐**2** . (b) VCD for unlabelled ALL and ADL. (c) VCD isotopologue overlay for ADL. (d) VCD isotopologue overlay for ALL. (e) Chiral integrity of unlabelled ALL after sodium salt formation and reacidification versus an original ALL sample.

With both labelled and unlabelled enantiomers to hand, we undertook a series of experiments to determine their stability, absolute configuration and stereochemical integrity. They were analysed using infrared (IR) and vibrational circular dichroism (VCD), which is sensitive to both chirality and isotopic substitution [25, 26, 27, 28, 29, 30]. As expected, the enantiomeric pairs, L‐ and D‐**2**, had similar IR spectra but mirror‐image VCD spectra (Figure 2a,b). Additionally, the VCD peak intensity relative to IR intensity for each band was the same for enantiomers; this showed that there was no significant epimerisation during synthetic transformations. The ^13^C‐substituted compounds had frequency shifts for many IR and VCD bands as compared to unlabelled compounds, yielding spectra with a significantly different appearance (Figure 2c and d). Despite this, there were some comparable bands between the two sets of spectra, and comparison of the VCD intensities showed that there was a similar enantiomeric excess (ee) in the isotopologue samples. Finally, L‐ and D‐**2** are usually formulated as their more soluble sodium salts for in vivo assays. We wished to investigate their stereochemical integrity [31] by recreating the formulation process by treatment with dilute NaOH, although we selected ALL as a cheaper test material. After being left overnight, starting material was recovered by re‐acidification and extracted for analysis to compare it to the original sample. This chirality integrity test, by VCD, established that the sample was still ALL without any epimerisation.

These findings were further confirmed by optical rotation and ellipticity measurements (see Experimental Section and Table 1), which show similar trends to those above, notably, virtually equal and opposite values for L‐ and D‐**2**. Finally, UHPLC (Ultra High Performance Liquid Chromatography) and SFC (Supercritical Fluid Chromatography) measurements allowed us to confirm high compound purity, further confirmed by 1H NMR in the presence of an internal standard, and ee for these compounds (using [racemic] ADLL as a control). Isotope incorporation had little effect, if any, on retention times (RT) in the final compounds, with the D‐enantiomer appearing around 3.27 min, whereas its L‐enantiomer appeared around 3.57 min. As ALL is used as its sodium salt in in vivo studies, we acidified and extracted a sample, and the recovered ALL, gratifyingly had a high chemical purity and ee.

**TABLE 1:** Ellipticity and chemical and optical purity for compounds.

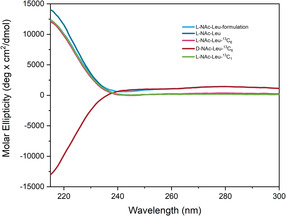						
Compound	Molar ellipticity (220 nm, deg·cm^2^/dmol)	% purity by ^1^H NMR^a^	UHPLC^b^ chemical purity, %	SFC chemical purity, %^c^	SFC RT, min	e.e, %
ALL	11 543	—	100 (m/z = 174.2)	98.3 (m/z = 174.1)	3.59	99.8
ALL recovered from Na salt	10 067	—	100 (m/z = 174.1)	98.3 (m/z = 174.1)	3.57	99.8
L‐**2**	9765	>95	99.5 (m/z = 180.2)	98.2 (m/z = 180.2)	3.57	97.8
D‐**2**	–9807	>95	100 (m/z = 180.2)	100 (m/z = 180.2)	3.27	100
L‐**4**	10 009	>95	100 (m/z = 175.2)	100 (m/z = 175.2)	3.57	100
ADL	—	—	100 (m/z = 174.2)	100 (m/z = 174.1)	3.27	99.8
ADLL	—	—	100 (m/z = 174.2)	99.2 (m/z = 174.1)	3.27/3.57	1.8

a Using a 1,3,5‐trimethoxybenzene internal standard.

b Acquity C_18_ HSS (2.1 × 50 mm, 1.8 μm), 45°C, 1 mL/min, 210 ‐400 nm detection. Mobile phase A (water (0.1% v/v TFA) and mobile phase B (MeCN).

c Chiralpak IG (4.6 × 250 mm, 5 μm), 40°C, 3 mL/min, 211 nm, 125 BarG (mobile phase A: MeOH (0.2% v/v NH_3_); mobile phase B: CO_2_.

## 3 Conclusions

Three acetylated ^13^C‐labelled leucine analogues, L‐ and D‐**2** and L‐**4**, have been synthesised and characterised by NMR spectroscopy and mass spectrometry. Products were studied by UHPLC, SFC, VCD, polarimetry and ellipticity measurements, which showed that they were stable enantiomers, even after salt formation, for in vivo assays. L‐**2** and D‐**2** are now being used as in vivo tool compounds in Niemann‐Pick disease type C1, and results of these studies will be published in due course. Furthermore, it is anticipated that compounds **2** and the more readily affordable **4** will be useful labelled tool compounds for studies in a much wider subset of neurological diseases [32, 33].

## 4 Experimental Section

^13^C‐labelled starting materials were purchased from Merck (L‐leucine‐^13^C_6_:605239, batch: MBBD0145). D‐leucine‐^13^C_6_: 921 416, batch: MBBD163. ^13^C_1_‐L‐leucine: 1 003 638 621, batch: MBBD6818) and used without further purification. NMR experiments were performed on a 600 MHz spectrometer equipped with a Varian 14 T magnet and a Bruker Avance Neo console at 25 °C. An HSQC spectrum was recorded using the hsqcedetgpsisp2.2 pulse programme with 64 scans per increment and 256 increments in the ^13^C dimension. Chemical shifts are quoted in parts per million (ppm) with coupling constants (*J*) in Hz. ESI mass spectra were obtained using a Waters Xevo G2 Q‐ToF HRMS (Wilmslow, UK) equipped with an analytical flow ESI source. ESI experimental parameters were capillary voltage 3.0 kV, sampling cone 35 au, extraction cone 4 au, source temperature 120°C and desolvation gas 450°C with a desolvation gas flow of 650 L h‐1 and no cone gas. MS conditions were MS1 in resolution mode between 100–1500 Da. Accurate mass data were obtained using MassLynx software. All accurate mass data were within ±10 ppm from their theoretical value. LC‐MS was performed on Shimadzu LC‐2050 and LCMS‐2020 systems using an 8‐minute method in water/acetonitrile with 0.1% formic acid (5% MeCN over 0.5 min, 5‐95% over 6.0 min, 95% MeCN over 1.0 min and 5% MeCN over 0.5 min) with UV detection at 254 nm with a Phenomenex Kinetex 2.6 μM EVO C18 100A LC Column (50 @× 3.0 mm). Unless stated otherwise, solvents and modifiers used were Fisher Scientific, Optima LC/MS Grade and Fisher Chemical. Optical rotations were performed either on an analogue WXG‐4 or digital AA‐10 polarimeter at 20°C temperature in EtOH, and concentrations were between 0.17–0.20 g/100 mL. Molar ellipticity was measured in a 1 cm cuvette on a Jasco J‐810 spectropolarimeter equipped with a thermostatted cuvette holder (at 20°C). Experimental parameters were: scan 215–300 nm, data pitch 1 nm, bandwidth 2 nm and scanning speed 100 nm/min.

### 4.1 VCD Measurements

To a small vial containing 4–5 mg of N‐Ac‐leucine (L or D, unlabelled or ^13^C) was added 185–225 μL of d_6_‐DMSO. The resulting solution was transferred to a liquid IR cell (BaF_2_, 100 μm cell path) and placed in the measurement chamber. The instrumentation was a BioTools Inc. (Jupiter, FL) ChiralIR 2X DualPEM FT‐VCD spectrometer, set to 4 cm^−1^ resolution, with PEM (both 1 and 2) maximum frequency set to 1400 cm^−1^. The sample was then measured for 8 in 1 h blocks. The IR data from the first block were solvent and water vapour subtracted, then offset to zero at 2000 cm^−1^. The VCD data blocks were averaged and the baseline corrected using solvent subtraction. Finally, the VCD spectrum was offset to zero at 2000 cm^−1^. The VCD noise data was block averaged and used without further processing.

#### 4.1.1 L‐2, N‐Acetyl‐L‐leucine‐^13^C_6_

L‐Leucine‐^13^C_6_ (750 mg, 5.47 mmol) was suspended in water (7.5 mL). The reaction mixture was cooled with an ice bath; then acetic anhydride (1.55 mL, 16.4 mmol) followed by a solution of sodium hydroxide (1.75 g, 43.8 mmol) in water (7.5 mL) was slowly added. The reaction mixture was stirred at room temperature for 3 h. Next, 2 M aqueous HCl (∼20 mL) was added up to pH ∼ 2–3. The reaction mixture was stirred at 0°C for 1 h, and the resulting white solid was collected by filtration, rinsing with water (2 × 10 mL). The solid was dried under vacuum at 40°C overnight to afford L‐**2** (890 mg, 4.72 mmol, 86% yield) as a colourless solid. ^1^H NMR (600 MHz, DMSO‐*d*_6_) was consistent with product structure at an estimated 95% purity with unlabelled carbons emboldened. ^1^H NMR (600 MHz, DMSO‐*d*_6_) *δ* 12.49 (s, 1H), 8.09 (d, *J* = 7.7 Hz, 1H), 4.36 – 3.99 (m, 1H), 1.83 (s, 3H), 1.76–1.27 (m, 3H), 1.04–0.88 (m, 3H), 0.82–0.68 (m, 3H). ^13^C NMR (151 MHz, DMSO‐*d*_6_) *δ* 174.3 (dd, *J* = 59.0, 2.9 Hz), **169.3** (faint signal), 50.2 (ddt, *J* = 59.4, 34.9, 2.8 Hz), 39.9 (t, *J* = 34.7 Hz), 24.3 (qd, *J* = 34.8, 3.0 Hz), 22.8 (dd, *J* = 34.8, 3.4 Hz), **22.4** (faint signal), 21.3 (dd, *J* = 34.8, 2.1 Hz). HRMS m/z [M+H]+ calculated for C^13^C_6_H_16_NO_3_: 180.1336; found 180.1335 (5 ppm). [*α*]_D_ = −30.1°. Molar ellipticity, 9765 deg·cm^2^/dmol (220 nm).

#### 4.1.2 D‐2, N‐Acetyl‐D‐Leucine‐^13^C_6_

D‐Leucine‐^13^C_6_ (95 mg, 0.69 mmol) was suspended in water (2 mL). The reaction mixture was cooled with an ice bath; then acetic anhydride (0.2 mL, 2.08 mmol) followed by a solution of sodium hydroxide (221 mg, 5.54 mmol) in water (1 mL) was slowly added. The reaction mixture was stirred at room temperature for 3 h. 2 M aqueous HCl (∼2.5 mL) was added up to pH ∼ 2–3. The reaction mixture was stirred at 0°C for 1 h, and the resulting colourless solid was collected by filtration, rinsing with water (3 × 2 mL). The solid was dried under vacuum at 40°C overnight to afford ^13^C_6_‐N‐acetyl‐D‐leucine (65 mg, 0.34 mmol, 50% yield) as a colourless solid. ^1^H NMR (500 MHz, DMSO‐*d*_6_) was consistent with product structure at an estimated 95% purity. ^1^H NMR (500 MHz, DMSO‐*d*_6_) *δ* 12.46 (s, 1H), 8.07 (d, *J* = 7.7 Hz, 1H), 4.42–3.96 (m, 1H), 1.83 (s, 3H), 1.79–1.28 (m, 3H), 1.06–0.91 (m, 3H), 0.81–0.66 (m, 3H).^13^C NMR (126 MHz, DMSO‐*d*_6_) *δ* 174.3 (dd, *J* = 59.3, 3.1 Hz), **169.2** (faint signal), 50.1 (ddt, *J* = 59.3, 34.8, 2.7 Hz), 40.1 (d, *J* = 34.6 Hz), 24.3 (qd, *J* = 34.5, 3.1 Hz), 22.8 (dd, *J* = 34.8, 3.2 Hz), **22.3** (faint signal), 21.3 (dd, *J* = 34.8, 2.2 Hz). HRMS m/z [M+H]+ calculated for C^13^C_6_H_16_NO_3_: 180.1337; found: 180.1326 (6.1 ppm). [*α*]_D_ was not measured due to the dilute solution, variability and small amount of material. Molar ellipticity, −9807 deg·cm^2^/dmol (220 nm).

#### 4.1.3 L‐4, N‐Acetyl‐L‐Leucine‐^13^C_1_

L‐Leucine‐^13^C_1_ (1.0 g, 7.57 mmol) was suspended in water (10 mL). The reaction mixture was cooled with an ice bath; then acetic anhydride (2.15 mL, 22.7 mmol) followed by a solution of sodium hydroxide (2.4 g, 60.5 mmol) in water (10 mL) was slowly added. The reaction mixture was stirred at room temperature for 3 h. 2 M aqueous HCl (∼30 mL) was added up to pH ∼ 2–3. The reaction mixture was stirred at 0°C for 1 h, and the resulting colourless solid was collected by filtration, rinsing with water (2 × 10 mL). The solid was dried under vacuum at 40°C overnight to afford ^13^C_1_‐N‐acetyl‐L‐leucine (700 mg, 3.82 mmol, 50% yield) as a colourless solid. ^1^H NMR (600 MHz, DMSO‐*d*_6_) was consistent with product structure at >95% purity. ^1^H NMR (600 MHz, DMSO‐*d*_6_) *δ* 12.49 (s, 1H), 8.10 (d, *J* = 8.0 Hz, 1H), 4.18 (ddt, *J* = 9.7, 7.9, 5.7 Hz, 1H), 1.83 (s, 3H), 1.66–1.57 (m, 1H), 1.53–1.41 (m, 2H), 0.88 (d, *J* = 6.6 Hz, 3H), 0.83 (d, *J* = 6.6 Hz, 3H). ^13^C NMR (151 MHz, DMSO‐*d*_6_) *δ* **174.3** (^13^C labelled, intense peak), 169.3, 50.2 (d, *J* = 59.4 Hz), 40.0, 24.3 (d, *J* = 3.2 Hz), 22.9, 22.3, 21.3. HRMS m/z [M+H]+ calculated for C_7_^13^CH_16_NO_3_: 175.1158 found to be 175.1162 (2.1 ppm). [*α*]_D_ = −24.5°. Molar ellipticity, 10 009 deg·cm^2^/dmol (220 nm).

### 4.2 Chiral Integrity Test; Re‐Hydrolysis of Na Salt of ALL


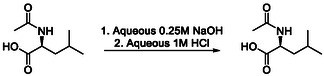


At room temperature (25°C), N‐acetyl‐L‐leucine (ALL, 100 mg, 0.58 mmol, [*α*]D = −23.9°, molar ellipticity = 11 543 (220 nm, deg·cm^2^/dmol)) was suspended in water (2.9 mL). The suspension was vortexed for 30 s then an aqueous solution of sodium hydroxide (0.25 M) (2.4 mL, 0.60 mmol) was added. The suspension was vortexed for 30 s to give a colourless solution. Water (0.5 mL) was added, and the reaction mixture was heated at 37°C for 24 h. At room temperature, an aqueous solution of hydrochloric acid (1.0 M) (0.6 mL, 0.60 mmol) was added. The solution was extracted with a 9:1 DCM/MeOH mixture (4 × 15 mL) then the combined organic layers were filtered through a phase separator. The filtrate was concentrated to dryness (Bucchi rotary evaporator bath temperature = 40°C). N‐Acetyl‐L‐leucine (70 mg, 0.38 mmol, 67% yield) was re‐isolated as a colourless solid. ^1^H NMR (500 MHz, DMSO‐*d*_6_) was consistent with product structure at an estimated 95% purity. ^1^H NMR (500 MHz, DMSO‐*d*_6_) *δ* 12.46 (s, 1H), 8.07 (d, *J* = 8.0 Hz, 1H), 4.19 (ddd, *J* = 9.4, 8.0, 5.8 Hz, 1H), 1.83 (s, 3H), 1.67–1.57 (m, 1H), 1.54–1.42 (m, 2H), 0.89 (d, *J* = 6.7 Hz, 3H), 0.84 (d, *J* = 6.5 Hz, 3H). [*α*]_D_ = −29.3°. Molar ellipticity = 10 067 deg·cm^2^/dmol (220 nm).

## Author Contributions

D.C., A.M., compound synthesis, characterisation, data analysis, manuscript writing. D.S., R.B, formulation studies, data analysis, manuscript writing. M.S. NMR studies and interpretation. J. N., VCD/IR studies, calculations, data analysis, manuscript writing. J.G.R., G.D.P., optical rotation, ellipticity studies. G.C., F.M.P.; project oversight, data analysis, funding acquisition. J.S; project oversight, funding acquisition, data analysis, manuscript writing.

## Funding

This study was supported by Wellcome Trust, Wolfson Society, Horizon 2020 Framework Programme (Lysomod MS‐C RISE 734825), Niemann‐Pick Research Foundation.

## Conflicts of Interest

The authors declare no conflicts of interest.

## Supporting information

Supplementary Material

## Data Availability

The ESI reports scans of NMR spectra and mass spectrometry results for all new compounds designed in this study.
