# Supplementary material for: Synthesis of N‐Acetyl‐D‐ and ‐L‐Leucine‐13C6 Tool Compounds in Neurodegenerative Disease
Source: ChemMedChem. 2026 May 15;21(9):e70295. doi: 10.1002/cmdc.70295 (PMC13179125; doi:10.1002/cmdc.70295)
Supplement: Supplementary file 1 — Supplementary Material [file CMDC-21-e70295-s001.pdf]

Synthesis of N-Acetyl-D- and -L-Leucine-$^{13}C_6$ Tool Compounds in Neurodegenerative Disease

Damien Crepin,[1,#] Andrew McGown,[1,2#] Dawn Shepherd,[3] Rebecca Braine,[3] Manvendra Sharma,[2] Jordan Nafie,[4] João Gabriel Ribeiro,[5] G. Dan Pantos,[5] Grant Churchill,[3*] Frances M Platt[3*] and John Spencer.[1,2*]

[1] *Sussex Drug Discovery Centre, School of Life Sciences, University of Sussex, Falmer BN1 9QJ, U.K.*

[2] *Department of Chemistry, School of Life Sciences, University of Sussex, Falmer BN1 9QJ, U.K.*

[3]*Department of Pharmacology, University of Oxford, Oxford OX1 3QT, U.K.*

[4]*BioTools Inc, 5730 Corporate Way; Suite #220, West Palm Beach, FL 33407 USA.*

[5] *Department of Chemistry, University of Bath, Claverton Down, Bath, BA2 7AY, U.K.*

[#] *joint first authors.*

Contents

NMR spectra for final compounds L- and D-**2**, L-**4**……………………………..……….Page S2

NMR spectra for chiral integrity of ALL *via* reacidification of Na salt………………….Page S5

Internal standard, purity (>95%) of L- and D-**2**….……………………………………….Page S6

Internal standard, purity (>95%) of L- **4**…………………………………………...….Page S7

HRMS data for L-, D-**2** and L-**4** …………………………………………………..…..Page S8

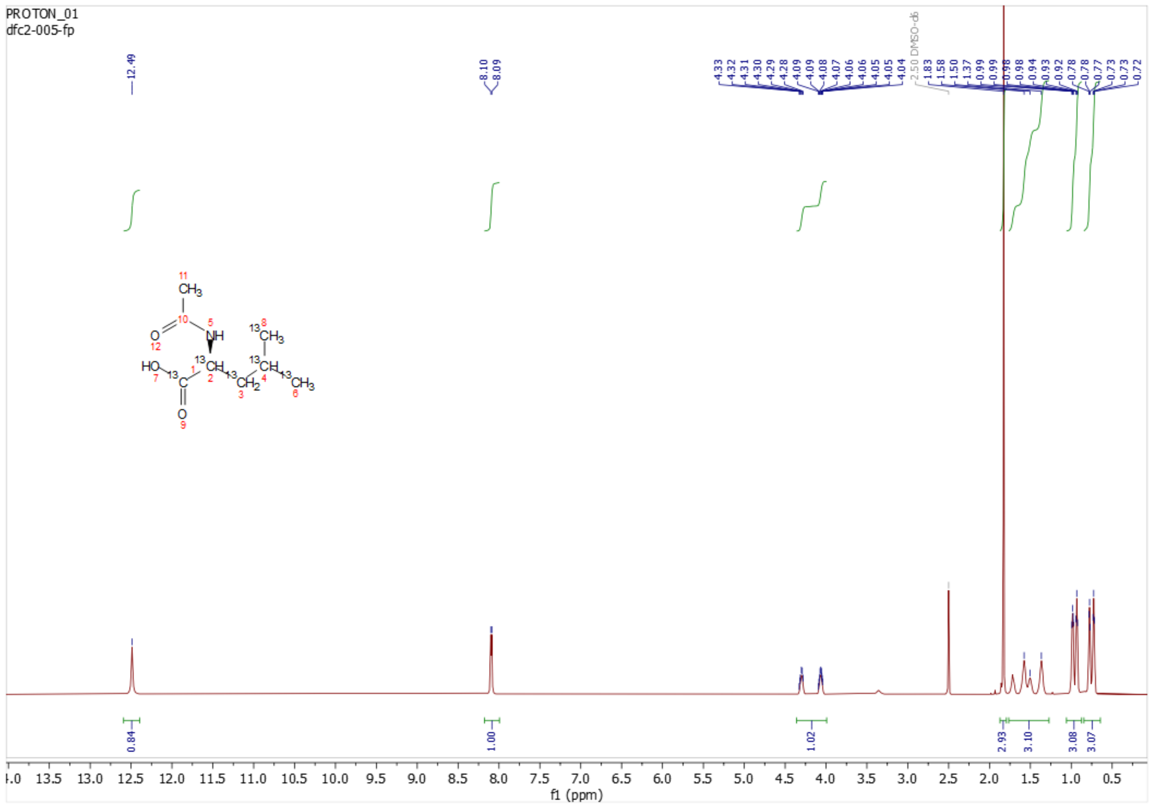
PROTON_01
dfc2-005-fp
12.49
8.10
8.09
4.33 4.32 4.31 4.30 4.29 4.28 4.09 4.09 4.08 4.07 4.06 4.06 4.05 4.05 4.04
2.50 DMSO-d6
1.83 1.58 1.50 1.37 0.99 0.99 0.98 0.98 0.94 0.93 0.92 0.78 0.78 0.77 0.73 0.73 0.72
0.84
1.00
1.02
2.93
3.10
3.08
3.07
f1 (ppm)

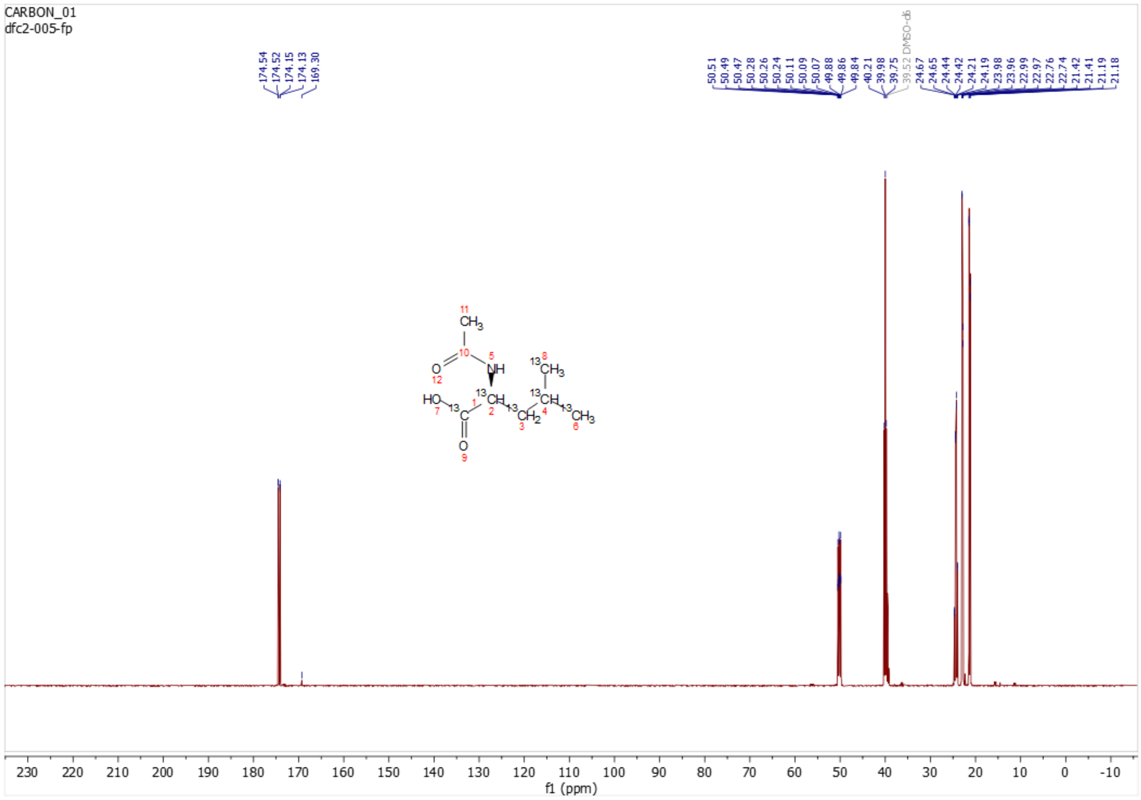
CARBON_01
dfc2-005-fp
174.54 174.52 174.15 174.13 169.30
50.51 50.49 50.47 50.28 50.26 50.24 50.11 50.09 50.07 49.88 49.86 49.84 40.21 39.98 39.75
39.52 DMSO-d6
24.67 24.65 24.44 24.42 24.21 24.19 23.98 23.96 22.99 22.97 22.76 22.74 21.42 21.41 21.19 21.18
f1 (ppm)

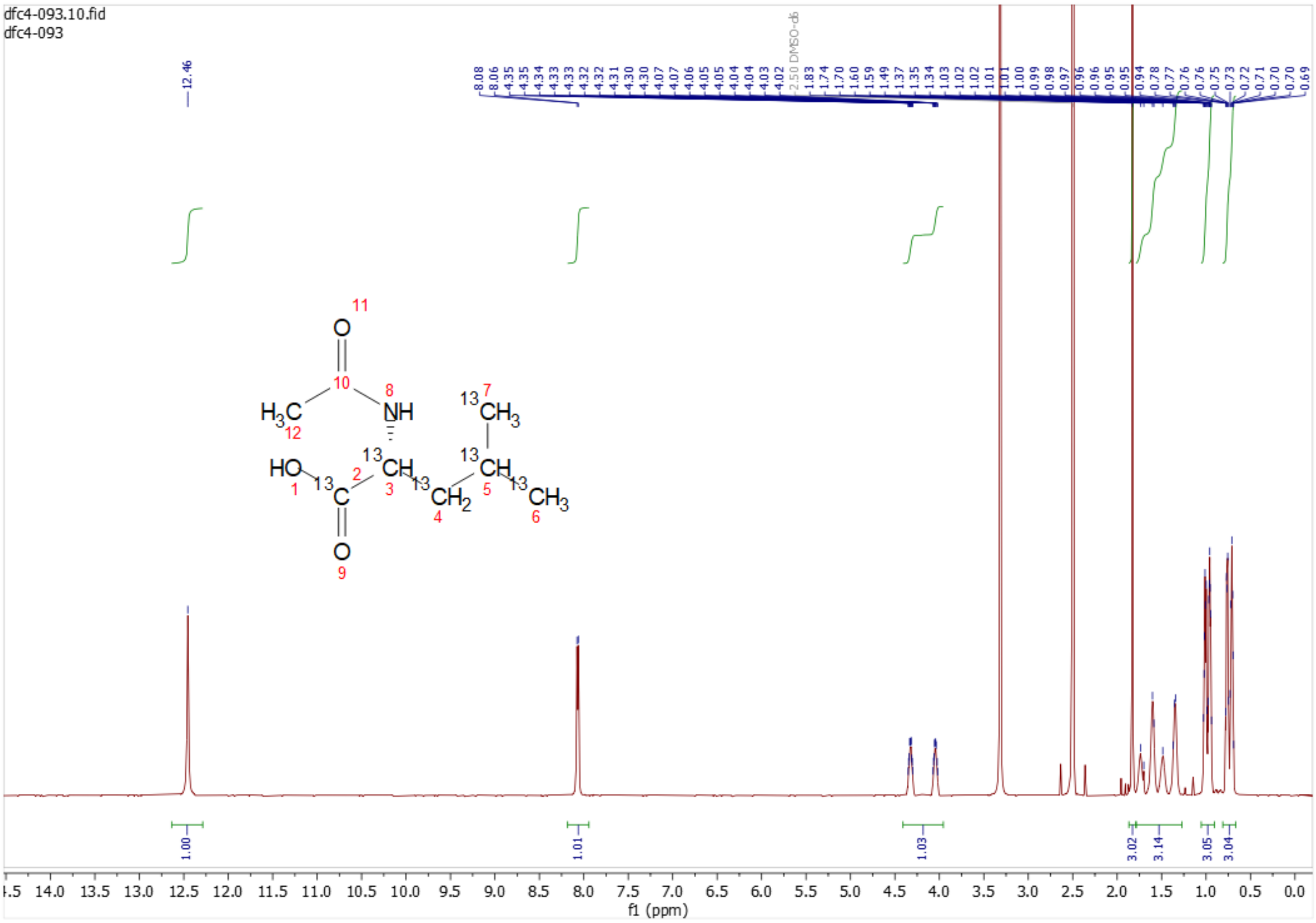

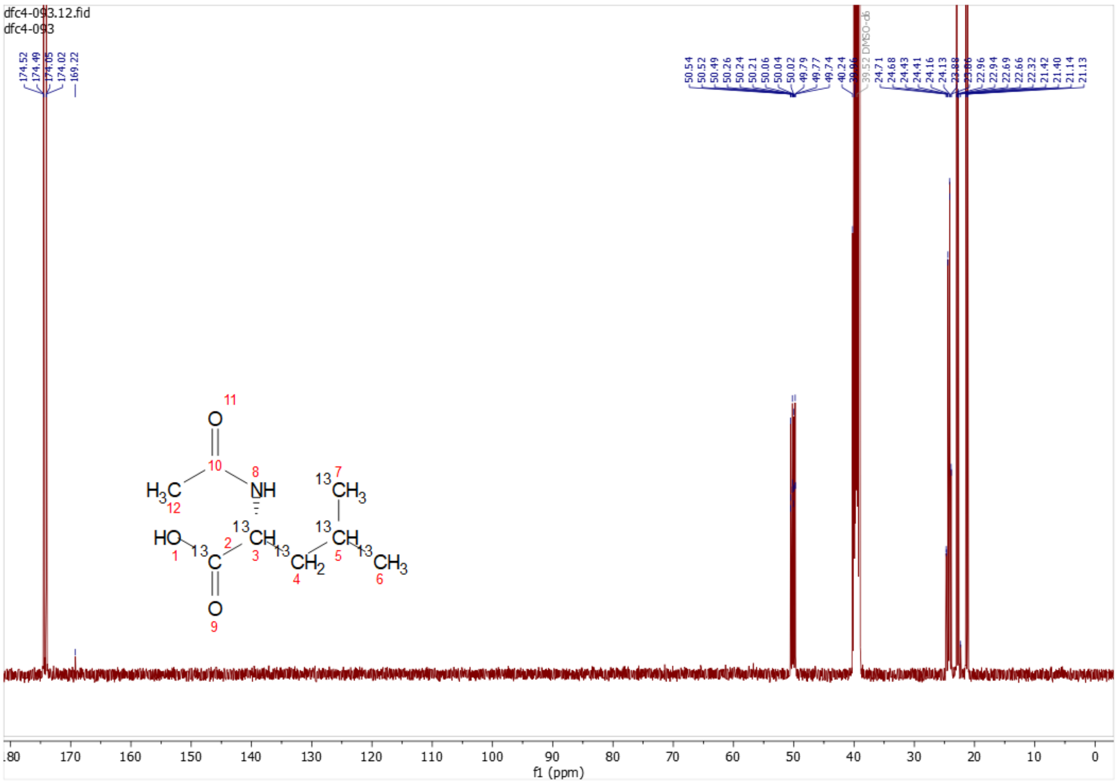

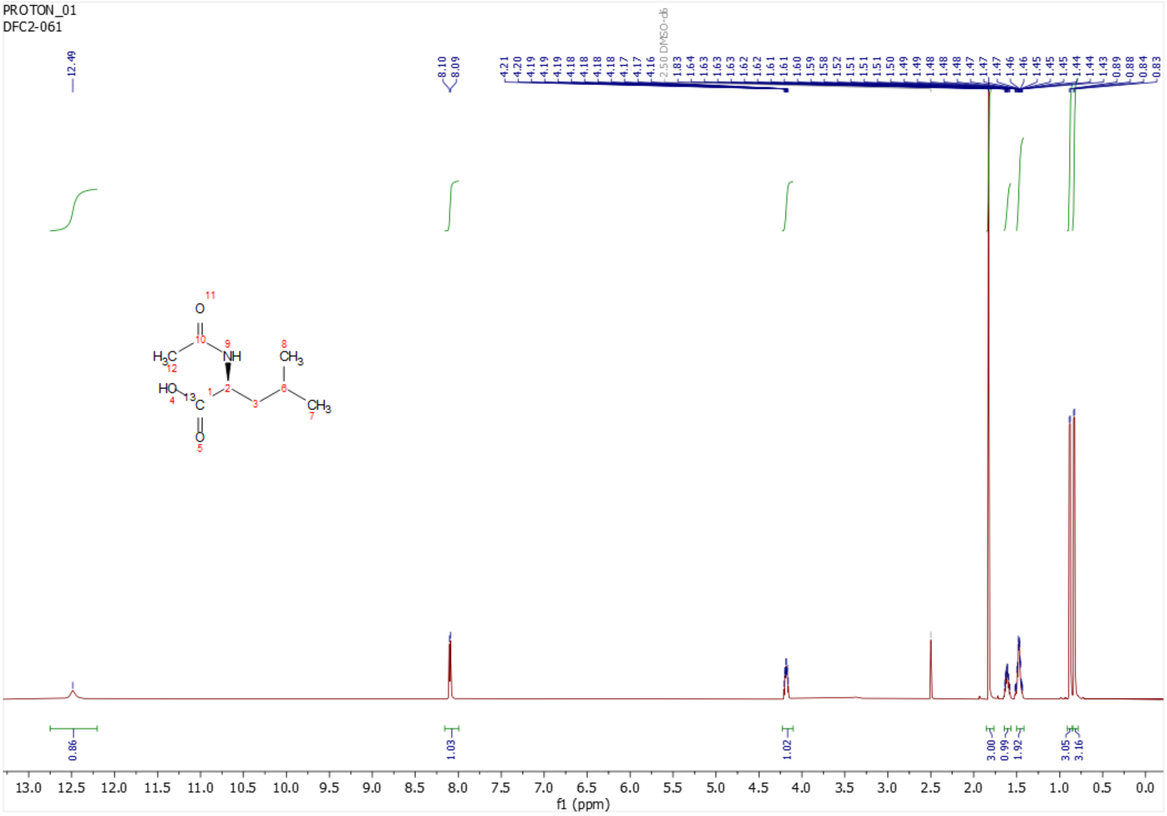
PROTON_01
DFC2-061
12.49
8.10
8.09
2.50 DMSO-d6
0.86
1.03
1.02
3.00
0.99
1.92
3.05
3.16
13.0 12.5 12.0 11.5 11.0 10.5 10.0 9.5 9.0 8.5 8.0 7.5 7.0 6.5 6.0 5.5 5.0 4.5 4.0 3.5 3.0 2.5 2.0 1.5 1.0 0.5 0.0
f1 (ppm)

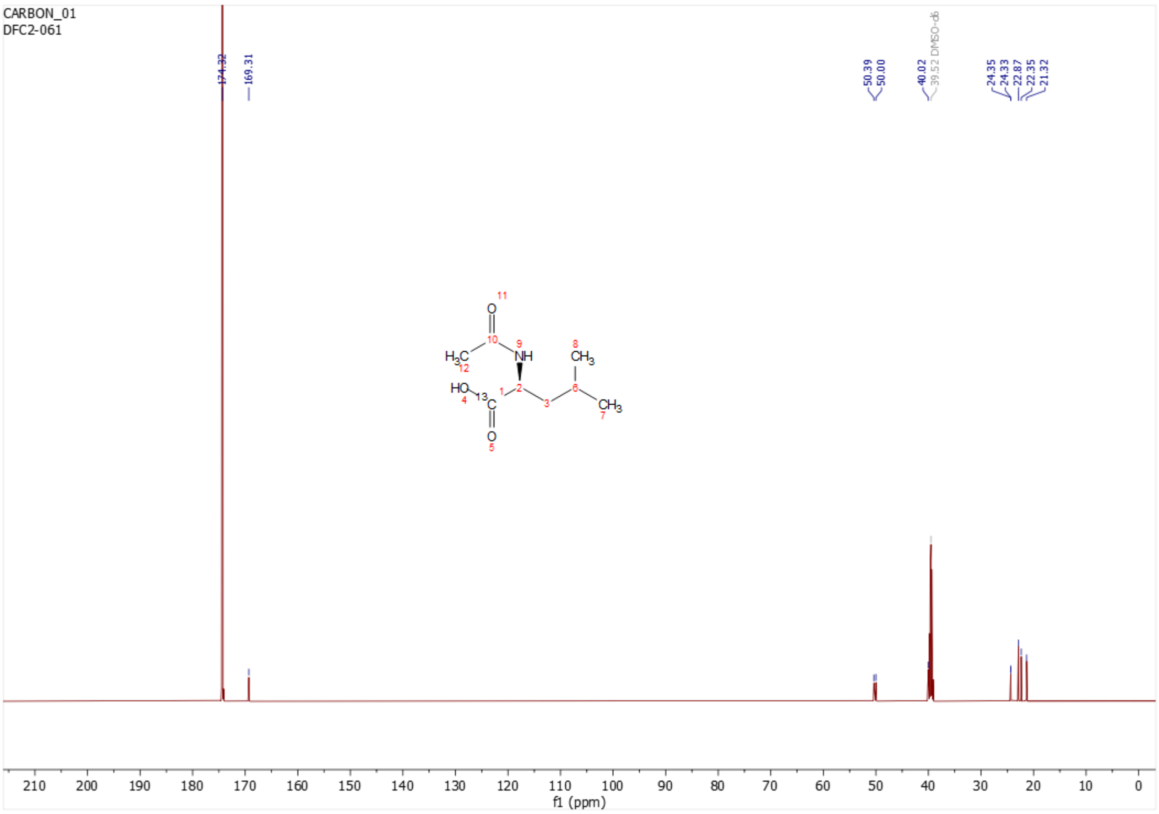
CARBON_01
DFC2-061
174.32
169.31
50.39
50.00
40.02
39.52 DMSO-d6
24.35
24.33
22.87
22.35
21.32
210 200 190 180 170 160 150 140 130 120 110 100 90 80 70 60 50 40 30 20 10 0
f1 (ppm)

Commercial ALL

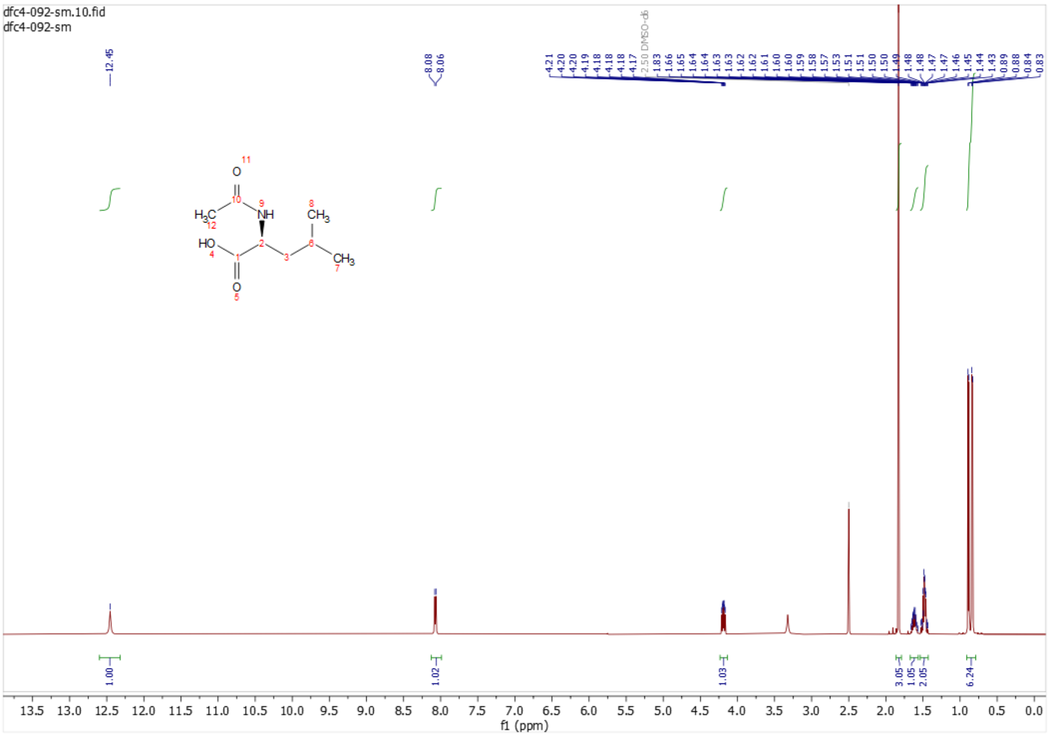

Recovered, after acidification of Na salt.

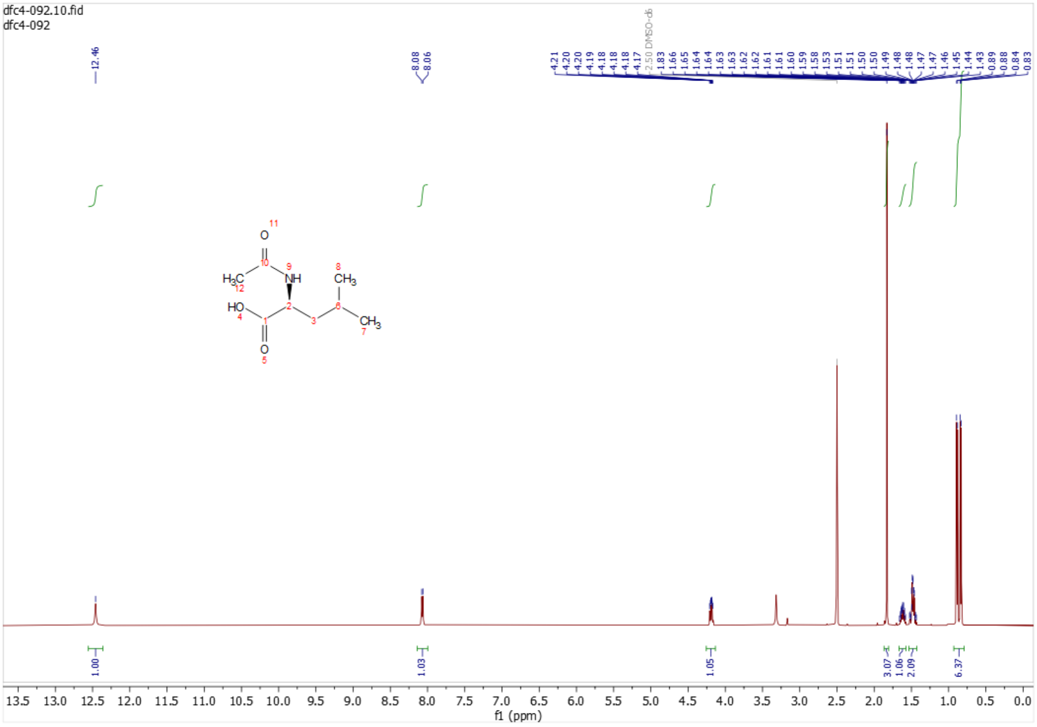

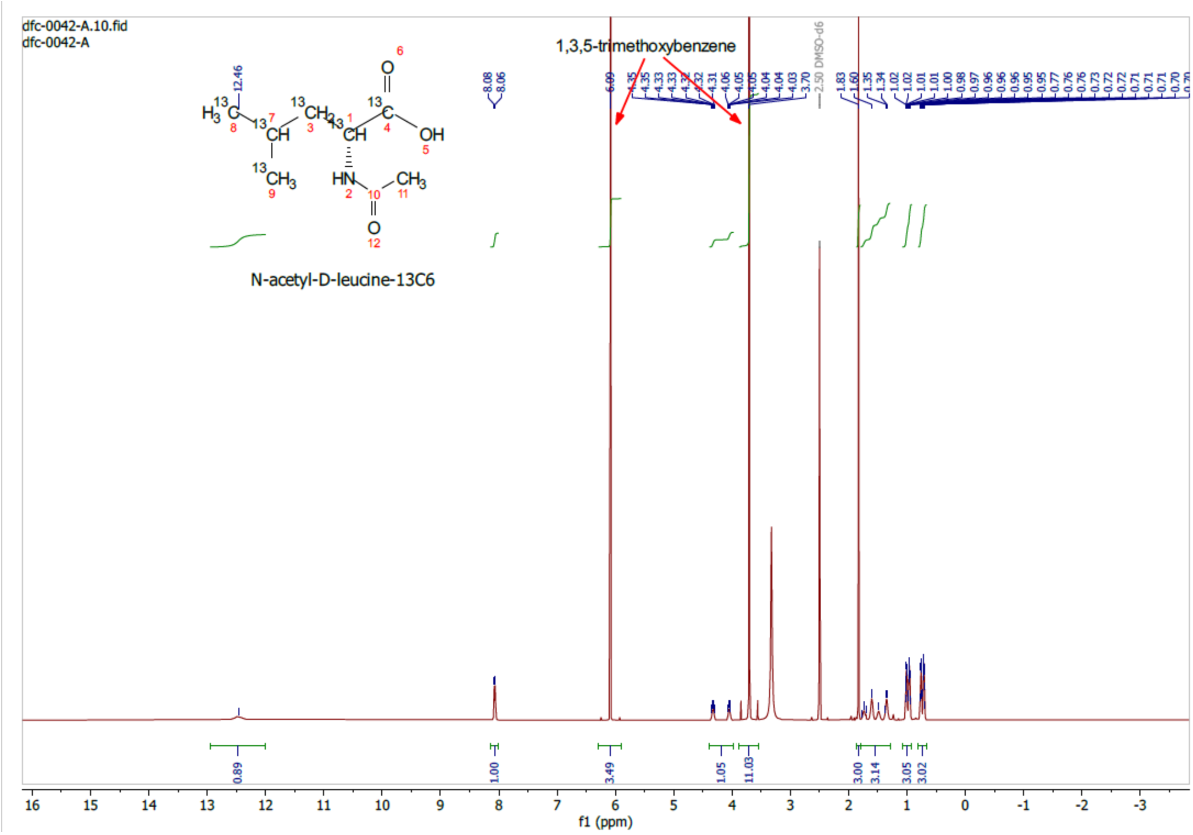

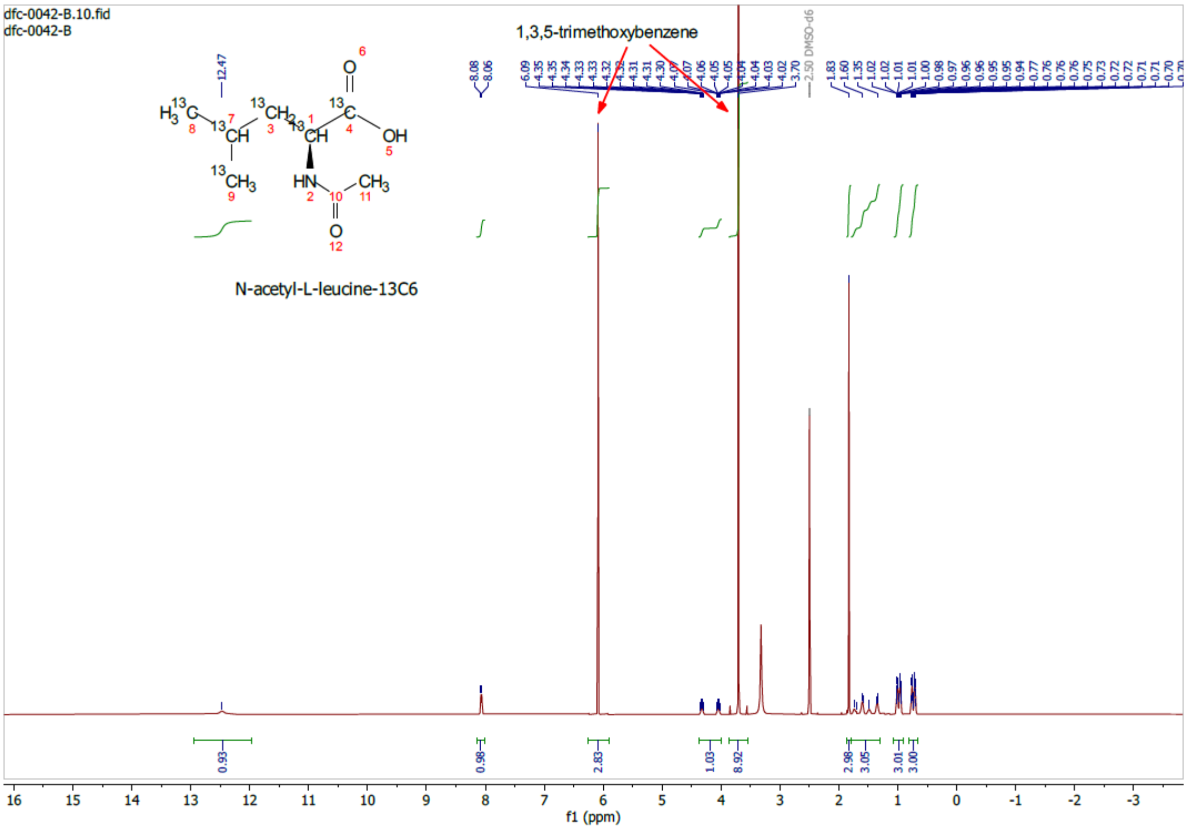

Purity for L- and D-**2** (>95%)

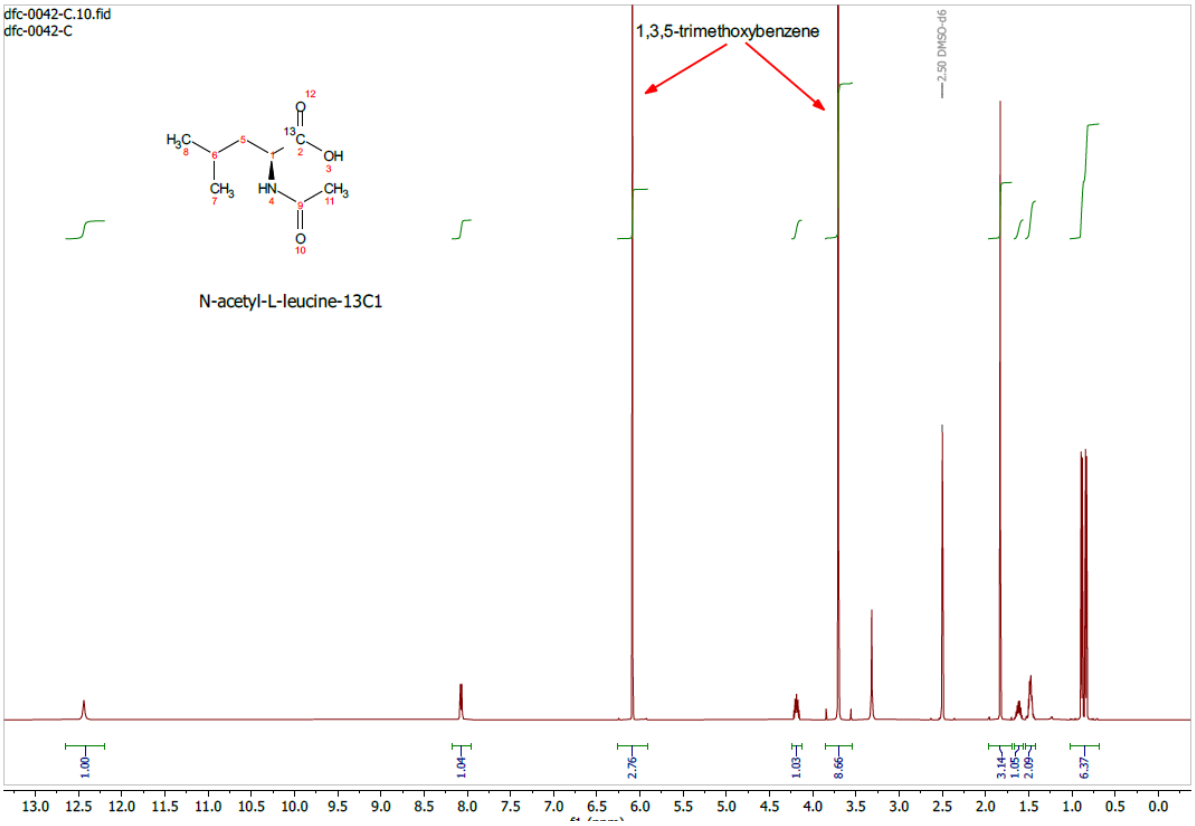

Purity for L-**4** (>95%)

(L)-**2**; HRMS

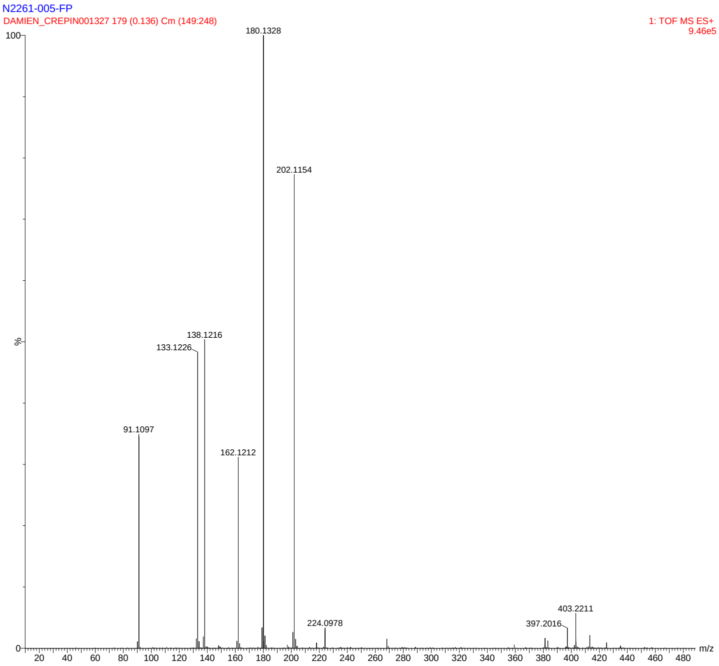

1) Mass error (in ppm):

Parameters:

Theoretical m/z: 180.1326

Observed m/z: 180.1335

Results:

Mass error: 4.996319 ppm

Calculate mass error

2) Theoretical m/z:

Parameters:

Main elements (monoisotopic):

C 2 H 16 N 1 O 3 S 0 P 0

Additional elements:

Na 0 Mg 0 F 0 K 0 Ca 0 Mn 0 Fe 0 Zn 0
I 0

Isotopes:

^{13}C 6 ^{2}H 0 ^{15}N 0 ^{18}O 0 ^{34}S 0 ^{35}Cl 0 ^{37}Cl 0

Charge +1 (Use negative values for negative ions)

Results:

Theoretical m/z: 180.132599

(D)-**2**; HRMS

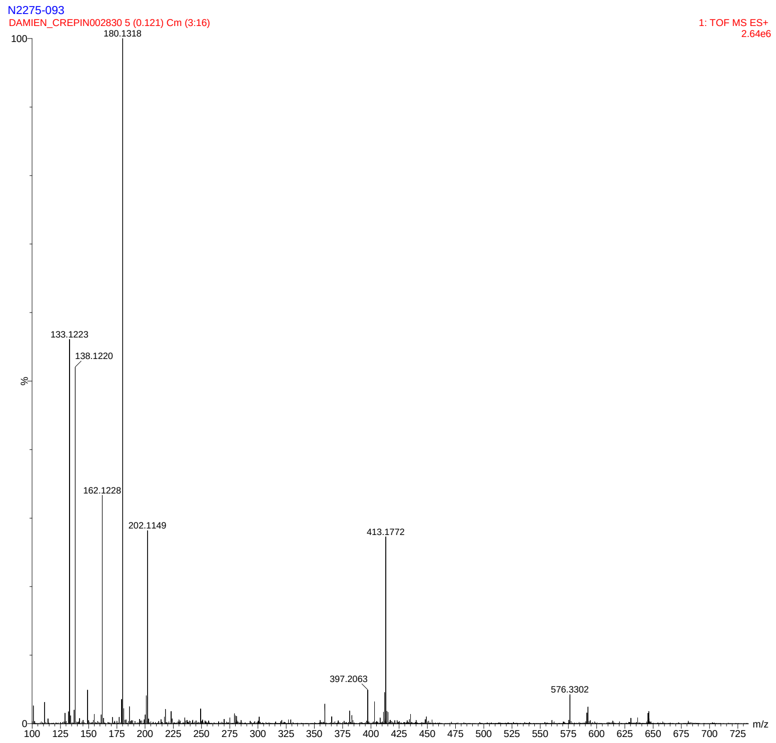

1) Mass error (in ppm):

Parameters:

Theoretical m/z: 180.133696

Observed m/z: 180.1326

Results:

Mass error: -6.084370 ppm

L-**4**; HRMS

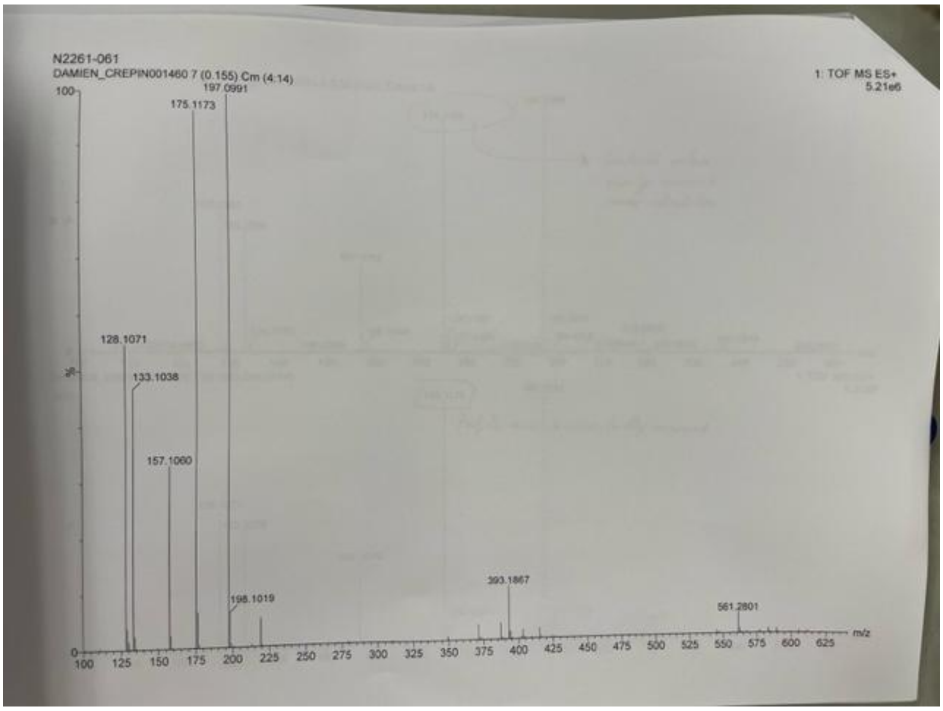

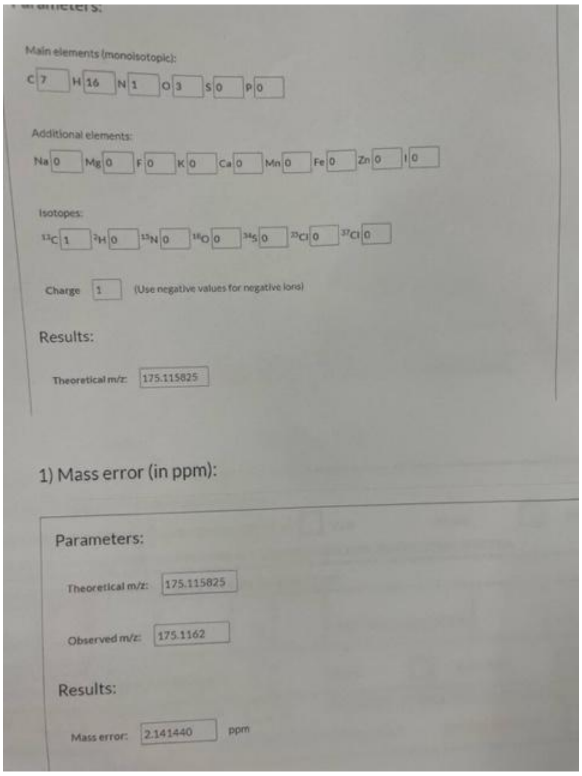
Main elements (monoisotopic):

C 7 H 16 N 1 O 3 S 0 P 0

Additional elements:

Na 0 Mg 0 F 0 K 0 Ca 0 Mn 0 Fe 0 Zn 0 I 0

Isotopes:

^{13}C 1 ^{2}H 0 ^{15}N 0 ^{18}O 0 ^{34}S 0 ^{35}Cl 0 ^{37}Cl 0

Charge 1 (Use negative values for negative ions)

Results:

Theoretical m/z: 175.115825

1) Mass error (in ppm):

Parameters:

Theoretical m/z: 175.115825

Observed m/z: 175.1162

Results:

Mass error: 2.141440 ppm

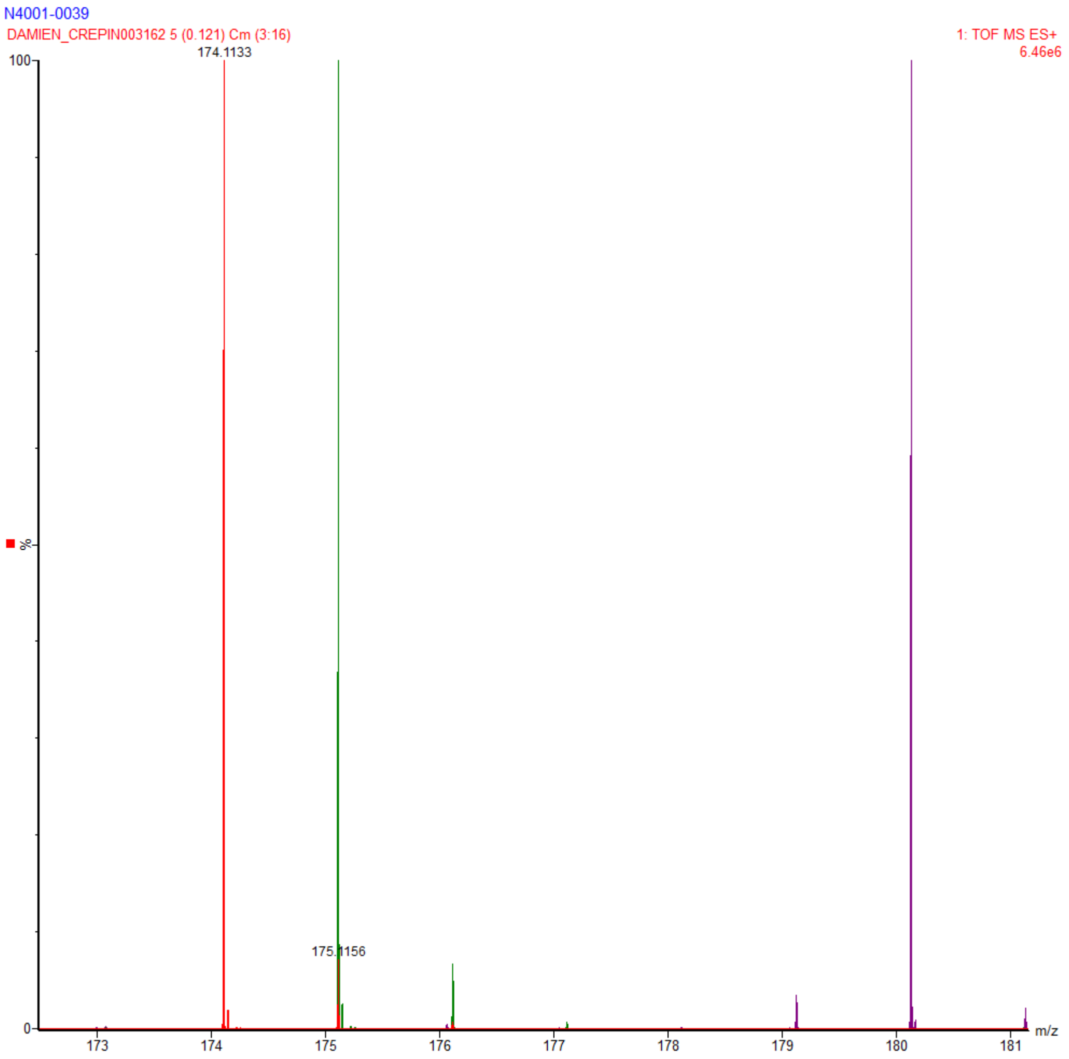

Mass spectra overlay
